# Overview of MicroRNAs as Diagnostic and Prognostic Biomarkers for High-Incidence Cancers in 2021

**DOI:** 10.3390/ijms231911389

**Published:** 2022-09-27

**Authors:** Chunyan Zhang, Caifang Sun, Yabin Zhao, Qiwen Wang, Jianlin Guo, Bingyu Ye, Guoying Yu

**Affiliations:** 1 State Key Laboratory Cell Differentiation and Regulation, Henan Normal University, Xinxiang 453007, China; 2 Henan International Joint Laboratory of Pulmonary Fibrosis, Henan Normal University, Xinxiang 453007, China; 3 Henan Center for Outstanding Overseas Scientists of Pulmonary Fibrosis, Henan Normal University, Xinxiang 453007, China; 4 College of Life Science, Henan Normal University, Xinxiang 453007, China; 5 Institute of Biomedical Science, Henan Normal University, Xinxiang 453007, China

**Keywords:** miRNAs, cancer, diagnosis, prognosis, biomarker

## Abstract

MicroRNAs (miRNAs) are small non-coding RNAs (ncRNAs) about 22 nucleotides in size, which play an important role in gene regulation and are involved in almost all major cellular physiological processes. In recent years, the abnormal expression of miRNAs has been shown to be associated with human diseases including cancer. In the past ten years, the link between miRNAs and various cancers has been extensively studied, and the abnormal expression of miRNAs has been reported in various malignant tumors, such as lung cancer, gastric cancer, colorectal cancer, liver cancer, breast cancer, and prostate cancer. Due to the high malignancy grade of these cancers, it is more necessary to develop the related diagnostic and prognostic methods. According to the study of miRNAs, many potential cancer biomarkers have been proposed for the diagnosis and prognosis of diseases, especially cancer, thus providing a new theoretical basis and perspective for cancer screening. The use of miRNAs as biomarkers for diagnosis or prognosis of cancer has the advantages of being less invasive to patients, with better accuracy and lower price. In view of the important clinical significance of miRNAs in human cancer research, this article reviewed the research status of miRNAs in the above-mentioned cancers in 2021, especially in terms of diagnosis and prognosis, and provided some new perspectives and theoretical basis for the diagnosis and treatment of cancers.

## 1. Introduction

Cancer is a global public health problem and a leading cause of death. As estimated by the WHO in 2019, cancer is the first or second leading cause of death before the age of 70 years in 183 countries around the world [1]. According to the GLOBOCAN 2020 data compiled by the International Agency for Research on Cancer (IARC), there will be 19,292,789 new cancer cases and 9,958,133 cancer deaths worldwide in 2020. Xia et al. conducted statistical analysis on cancer development in China over the years based on data from the GLOBOCAN 2020, the UN world Population Outlook 2019 (Revised Edition), the National Cancer Center of China (NCC) and other websites. According to their results, the incidence and mortality of colorectal cancer (CRC), female breast cancer (BC), and male prostate cancer (PCa) have significantly increased. It is predicted that the top five cancer types in China in 2022 will be lung cancer, CRC, gastric cancer (GC), liver cancer, and BC [2]. The incidence and mortality of cancer show an increasing trend year by year. The early diagnosis of cancer contributes to better patient treatment, markedly improved prognosis, reduced risk of cancer recurrence, and partially decreased cancer patient mortality.

The commonly used methods for the diagnosis and prognosis of cancers are (1) imaging examination: including X-ray, CT, magnetic resonance imaging (MRI), ultrasound, endoscopy, glucose metabolism technology and positron emission tomography (PET), radionuclide imaging examination and other imaging methods [3,4,5]; (2) molecular marker examination: including detection of tumor markers such as carcinoembryonic antigen (CEA), alpha-fetoprotein (AFP), and various carbohydrate antigens (such as CA125 and CA19) and some tumor-related biochemical indicators such as acid phosphatase (ACP), estrogen receptor (ER), and progesterone receptor (PR) in serum urine of patients [4,6,7,8,9]; (3) pathological examination where the abnormal tissue samples are collected for pathological examination by techniques such as immunohistochemistry (IHC), HE staining, and fluorescence in situ hybridization (FISH) [10,11]; (4) detection of circulating tumor cells (CTC), such as detection of tumor cells in peripheral blood, which can be used to monitor and predict the prognosis of tumor metastasis [12,13]; (5) body fluid cytological diagnosis, including detection of tumor cells by sputum, urine and other liquids or by means of puncture [14,15]; and (6) other examinations: including digital rectal examination (DRE), fecal occult blood test (FOBT), and other diagnostic methods [16,17].

**Figure 1:**
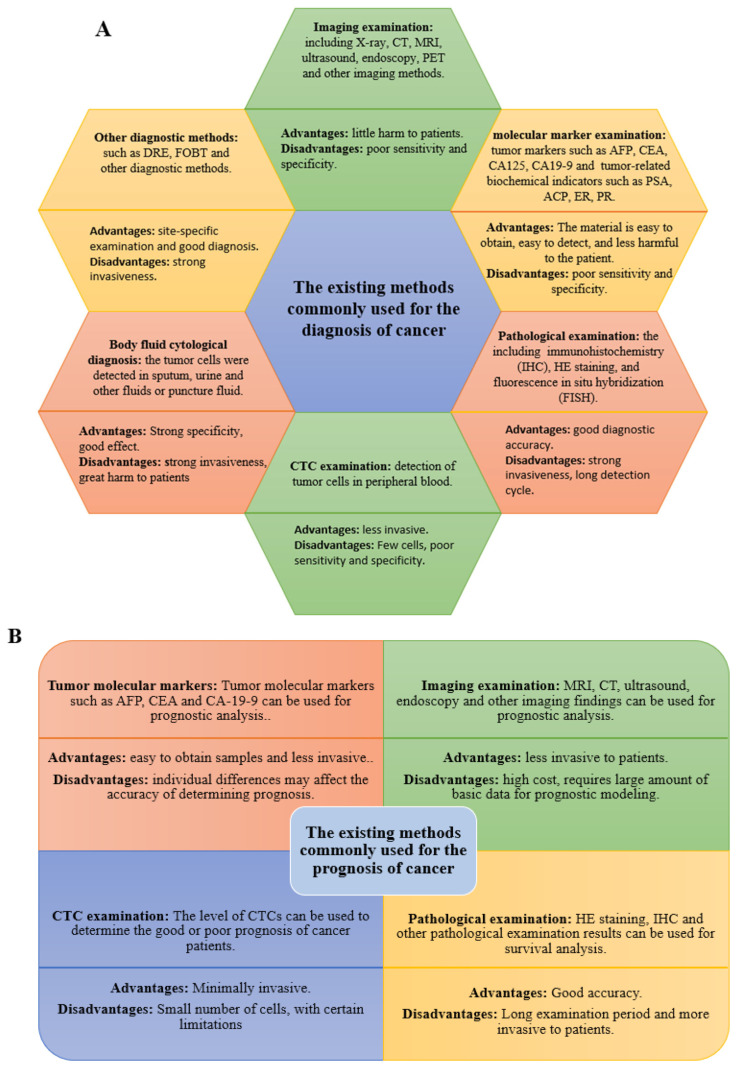
(**A**) The commonly used methods for the diagnosis of cancer. (**B**) The commonly used methods for the prognosis of cancer.

However, these diagnostic methods are either invasive and expensive, or show poor sensitivity and specificity. For example, colonoscopy for CRC diagnosis and gastroscopy for GC diagnosis will bring extreme discomfort to patients, and making pathological sections will cause greater trauma to patients, the pathological tissue for pathological sections needs to be obtained by biopsy, and the location and size of the samples will affect the test results, which will cause discomfort and even trauma to the patients [10,18]. While the other less invasive detection methods such as ultrasound, MRI, and serum tumor markers CEA, AFP, and other tests exhibit lower sensitivity or specificity [4,18]. Therefore, it is necessary to develop more convenient, rapid, and sensitive diagnostic and prognostic methods. The existing methods commonly used for the diagnosis and prognosis of cancer are summarized in Figure 1.

miRNAs are the non-coding RNAs (ncRNAs) that have been widely investigated in tumors recently. miRNAs are found to play an important role in the diagnosis and prognosis of cancer [19,20,21]. Therefore, the use of miRNAs as biomarkers for cancer diagnosis and prognosis has gradually attracted wide attention from people. In view of the 2021 papers regarding the relationship between miRNAs and different cancers retrieved in NCBI, the GLOBOCAN 2020 data, and the analysis and prediction of cancer development trends in recent years by Xia et al., this article selected six cancer types with high incidence, including GC, CRC, lung cancer, BC, PCa, and liver cancer, and summarized and analyzed the research progress of miRNAs as biomarkers for their diagnosis and prognosis.

## 2. Biosynthesis and Functions of miRNAs

The biosynthesis of miRNA is accomplished by a variety of enzymes. First, primary miRNAs (Pri-miRNAs) are transcribed under the action of RNA polymerase II, and then precursor miRNAs (pre-miRNAs) are formed under the action of RNase III Drosha and its cofactor DGCR8. Then it is cleaved into double-stranded miRNAs by endonuclease Dicer, and finally the mature single-stranded miRNAs are dissociated by helicase [22,23,24]. In addition, there is another miRNA biosynthesis pathway independent of the Drosha/DGCR8 pathway, which is called miRtrons, miRNA is processed by introns of protein-coding genes through mRNA pre-splicing mechanism in this pathway [25].

**Figure 2:**
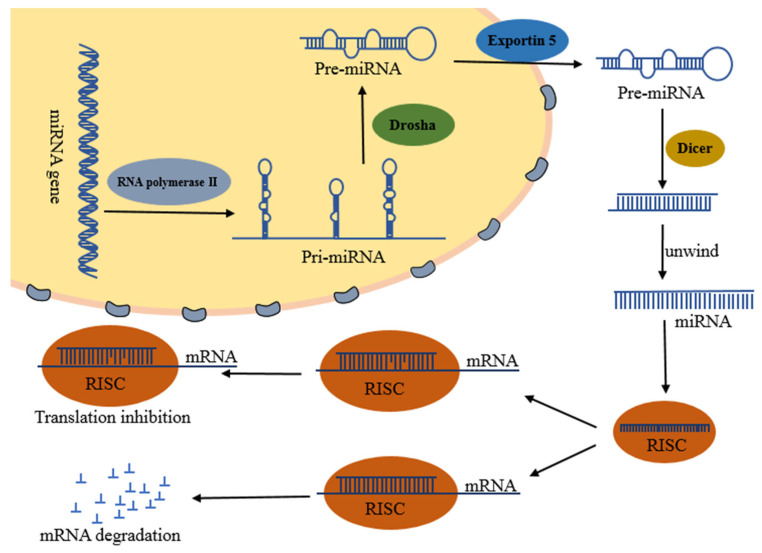
miRNA formation and mechanism of action.

In general, miRNAs regulate target genes at the post-transcriptional level, and destabilize the target mRNA or inhibit the translation of target mRNA [26]. According to their different modes of action with genes, they can be divided into the following three types [27,28,29,30], namely, ① complementary binding with target gene, which shares extremely similar mode of action and function to siRNA, and finally cleaves mRNA. This is a common mode in plants. ② Incomplete complementary binding with target gene, which prevents translation without affecting the stability of mRNA. It is the most commonly found mode of action and is common in animals. ③ The combination of the above two modes of action. Figure 2 summarizes the miRNA formation and mechanisms of action.

## 3. miRNAs and Cancer Diagnosis and Prognosis

**Figure 3:**
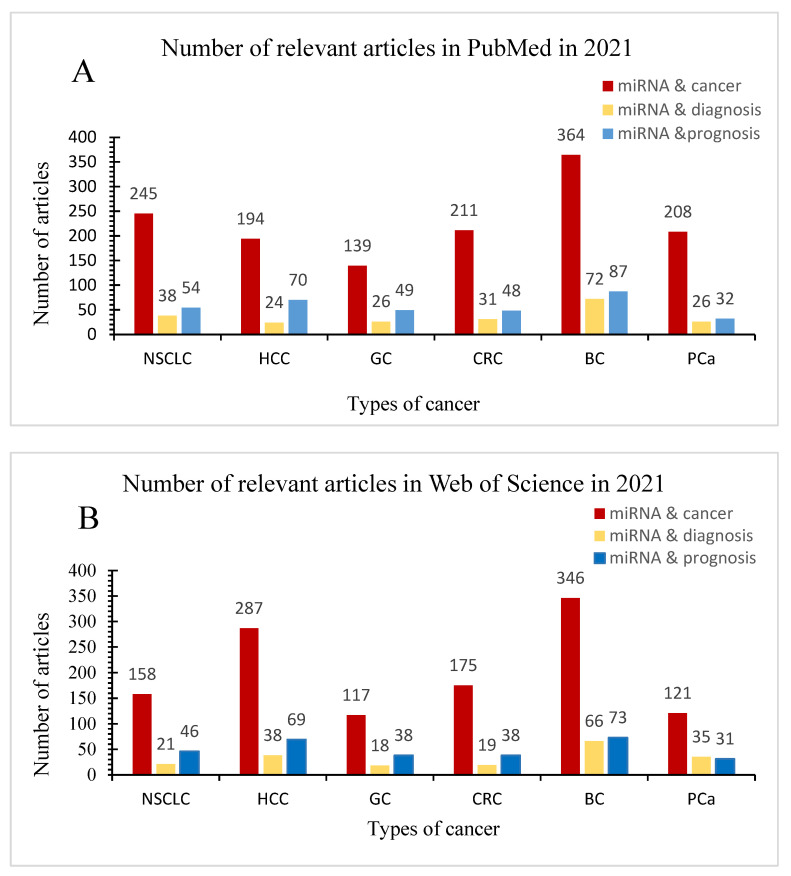
Articles on the diagnosis and prognosis of miRNAs in six cancers in 2021. (**A**) Number of relevant articles in PubMed in 2021. (**B**) Number of relevant articles in web of Science in 2021.

In recent years, miRNAs have attracted the attention of many researchers as biomarkers for cancer diagnosis and prognosis. In order to more systematically understand the research and application of miRNAs in the diagnosis and prognosis of these six cancers in 2021, we searched related papers in PubMed and Web of Science by combining miRNA, diagnosis, and prognosis with lung cancer, colorectal cancer, gastric cancer, liver cancer, breast cancer, and prostate cancer respectively. Articles on the diagnosis and prognosis of miRNAs in six cancers in 2021 are summarized in Figure 3.

Tumor is essentially a multi-gene abnormal disease. Its malignant phenotype is the result of the activation of expression of one or more proto-oncogenes or the loss of mutation of tumor suppressor genes, which enables tumor cells to escape from the normal growth regulation mechanism and to proliferate and invade autonomously. Due to the wide variety of miRNAs, they play different roles in different cancers, suggesting the higher specificity of miRNAs. Therefore, we reviewed the roles of miRNAs according to the characteristics of cancer types.

### 3.1. Lung Cancer

Lung cancer is a highly malignant cancer, which ranks the second place in terms of its morbidity and mortality in the world, accounting for 11.6% of all new cancer cases and 19.8% of all cancer-related deaths. Typically, non-small cell lung cancer (NSCLC) occupies 80% of lung cancer cases [31]. Recently, great progress has been made in identifying novel diagnostic and prognostic biomarkers for lung cancer, including RBR E3 ubiquitin ligase [32], prostaglandin E synthase 3 (PTGES3) [33], fibroblast activation protein (FAP) [34], checkpoint inhibitor immunotherapy targeting programmed cell death 1 (PD1), and programmed death ligand 1 (PDL1) pathways [35,36]. However, the 5-year overall survival rate of lung cancer remains low among all the cancer types, and its high mortality can be ascribed to the fact that most patients are diagnosed at an advanced stage and the treatment options are thereby limited, with a 5-year survival rate of only 4% [37]. The presence of reliable biomarkers can detect lung cancer at an early stage and can improve prognosis and recurrence risk. Several recent studies have demonstrated that miRNAs can be used as the diagnostic and prognostic biomarkers for lung cancer. The miRNAs associated with lung cancer identified in 2021 are summarized in Table 1.

Early diagnosis is the key to the successful treatment of lung cancer. As revealed by multiple human studies, miRNAs may serve as the tools for the early diagnosis of lung cancer. For example, Monika Migdalska-Sęk et al. found that miRNA-17 was up-regulated in NSCLC tissue and its surrounding para-cancerous tissues, so miRNA-17 was speculated to have an oncogenic effect. In addition, the expression of miRNA-17 in lung adenocarcinoma (LUAD), lung squamous cell carcinoma (LUSC), and the two NSCLC subtypes was significantly different [38]. miRNAs can also be used to diagnose NSCLC metastasis. Leptomeningeal metastasis (LM) is the most serious complication in NSCLC patients. The survival time of patients will be greatly shortened once LM occurs. Xu et al. found that the levels of miRNA-483-5p and miRNA-342-5p in serum exosomes of LM patients were higher than those of NSCLC patients without LM. Therefore, miRNA-483-5p and miRNA-342-5p might replace the routine examination of cerebrospinal fluid (CSF) to diagnose the occurrence of LM in NSCLC patients [39], which greatly reduced the trauma caused by CSF examination to patients. For the screening of miRNAs biomarkers for lung cancer, bioinformatics methods can also be used apart from using samples such as serum and tissues. Nie et al. analyzed the miRNAs expression profiles of lung cancer by bioinformatics analysis and discovered that miRNA-708-5p was significantly up-regulated in NSCLC tissues. To understand its sensitivity and specificity, a receiver operating characteristic (ROC) curve was plotted, and the area under the curve (AUC) was ≥0.9, indicating that miRNA-708-5p exhibited a good diagnostic value for NSCLC [40]. In addition, carcinoembryonic antigen (CEA) as a diagnostic marker for tumor has been widely used in clinical practice, but it has some shortcomings such as poor specificity. Therefore, the combination of CEA and other markers is among the popular cancer detection methods at present. Dong et al. combined CEA with serum miRNA-1247-5P, miRNA-301B-3p, and miRNA-105-5P, respectively, for the early diagnosis of NSCLC. Compared with miRNA alone, the AUC value of CEA combined with serum miRNAs increased from 0.812, 0.788, and 0.793 to 0.900, 0.895, and 0.904, respectively [41]. These results indicated that the combination of multiple markers for the diagnosis of NSCLC outperformed the use of miRNA alone, and the diagnostic effect was greatly improved, enhancing the detection accuracy.

In addition to diagnosis, miRNAs can also serve as the prognostic biomarkers for lung cancer. Similar to other malignancies, tumor stage is the most important determinant of lung cancer prognosis along with other clinical and histological variables [42]. In recent years, miRNAs have been considered as the key factors for predicting cancer prognosis, including lung cancer [43,44,45]. Yang et al. suggested that miRNA-1276, miRNA-767-3p, and miRNA-767-5p were up-regulated in LUAD by bioinformatics approaches, and the up-regulated expression of miRNA-1276 was negatively correlated with patient prognosis, whereas the up-regulated expression of miRNA-767-3p and miRNA-767-5p was negatively correlated with the survival time of patients. At the same time, Yang et al. verified the above bioinformatics results in two lung cancer cell lines A549 and SK-MES-1 [46]. As indicated by Khandelwal et al., the expression of miRNA-320a in the serum of NSCLC patients was significantly down-regulated. Moreover, its expression was correlated with the prognosis of NSCLC patients, and patients with low miRNA-320a expression had significantly decreased survival [47]. In addition, miRNAs can also be used for personalized studies in LUAD patients. Boldrini et al. analyzed miRNAs in 88 LUAD specimens and demonstrated that miR-25 expression was up-regulated in young LUAD patients compared with older LUAD patients, consistent with the results obtained from TCGA database. Furthermore, mechanism research showed that high expression of miR-25 was associated with the low expression of phosphatase and tensin homolog (PTEN) in young patients [48]. This study revealed the differential expression of miRNA-25 in young and elderly LUAD patients, and in particular, the interaction of miRNA-25 and PTEN in young LUAD patients may define a subgroup, highlighting the concept of molecular detection of different age subtypes. It provided a theoretical basis for whether lung adenocarcinoma (LUAD) patients of different ages have different molecular characteristics, and provided a preliminary guarantee for appropriate miRNA individualized treatment of LUAD. It also provided a theoretical basis and direction for the screening of tumor diagnostic and prognostic markers in the future, which should consider whether individual differences of patients (such as age, gender, and the presence of other diseases) will affect the screening results.

**Table 1:** MicroRNAs as biomarkers for diagnosis and prognosis of NSCLC.

miRNA	Sample	Expression	AUC	Biomarker	Reference
miR-708-5p	Tissue	↑	0.925	Diagnosis	[40]
miR-1247-5p	Serum	↑	0.812	Diagnosis	[41]
miR-301b-3p	Serum	↑	0.788	Diagnosis	[41]
miR-105-5p	Serum	↑	0.793	Diagnosis	[41]
miR-492	Plasma	↑	3-miR:0.828	Diagnosis	[49]
miR-590-3p	Plasma	↑	3-miR:0.828	Diagnosis	[49]
miR-4507	EBC	↑	3-miR:0.837	Diagnosis	[50]
miR-451a	EBC	↑	3-miR:0.837	Diagnosis	[50]
miR-483-5p	exosomes	↑	/	Diagnosis	[39]
miR-342-5p	exosomes	↑	/	Diagnosis	[39]
miR-520c-3p	exosomes	↑	2-miR:0.857	Diagnosis	[51]
miR-1274b	exosomes	↑	2-miR:0.857	Diagnosis	[51]
miR-96	exosomes	↑	0.9735	Diagnosis/Prognosis	[52]
miR-1276	Tissue	↑	/	Prognosis	[46]
miR-767-3p	Tissue	↑	/	Prognosis	[46]
miR-767-5p	Tissue	↑	/	Prognosis	[46]
miR-10b	Tissue	↑	/	Prognosis	[53]
miR-135b	Tissue	↑	/	Prognosis	[54]
miR-142-3p	Tissue	↑	/	Prognosis	[55]
miR-196a	Tissue	↑	/	Prognosis	[56]
miR-202	Plasma	↑	/	Prognosis	[57]
miR-17	Tissue	↓	0.692	Diagnosis	[38]
miR-125b-5p	Tissue	↓	0.768	Diagnosis/Prognosis	[44]
miR-631	Plasma	↓	3-miR:0.828	Diagnosis	[49]
miR-6777-5p	EBC	↓	3-miR:0.837	Diagnosis	[50]
miR-23a	exosomes	↓	0.744	Diagnosis	[58]
miR-let7i	exosomes	↓	0.733	Diagnosis	[58]
miR-203	Tissue	↓	/	Prognosis	[53]
miR-320a	Serum	↓	/	Prognosis	[47]

EBC: exhaled breath condensate, 3-miR: AUC values of three miRNA combinations appearing in the same paper, 2-miR: AUC values of two miRNA combinations appearing in the same paper, ↑: Up-regulated expression, ↓: Down-regulation expression.

### 3.2. Liver Cancer

Liver cancer is the fourth leading cause of cancer death worldwide. There are two subtypes of liver cancers, including hepatocellular carcinoma (HCC) and cholangiocarcinoma, of which HCC accounts for 80% [59]. Based on liver cancer data from 204 countries from 1990 to 2019, viral hepatitis B and C are found to be the leading cause of death from liver cancer [60]. Although great progress has been made in the treatment of HCC in recent decades, the 5-year survival rate of HCC patients remains as low as about 20%. This is mainly ascribed to the delayed diagnosis of HCC, chemotherapy resistance, frequent recurrence, and poor patient prognosis [61]. Consequently, the accurate diagnosis of HCC is important, which will improve the therapeutic effect and survival rate of patients. More recent studies have indicated that miRNAs may serve as the diagnostic and prognostic biomarkers for HCC. Notably, circulating miRNAs in serum or exosomes may be more useful biomarkers than those in tissues, since blood samples can be collected repeatedly in a non-invasive manner and show high specificity. miRNAs associated with HCC identified in 2021 are summarized in Table 2.

Early detection of HCC at a surgically resectable stage provides the best chance of survival for patients, and it is needed to find effective biomarkers for detecting HCC and tracking its development. It has been indicated in numerous studies that miRNAs can potentially serve as the tools for the early diagnosis of HCC. For example, Qin et al. performed miRNAs differential expression analysis on TCGA dataset and the miRNAs expression profiling data of 48 HCC patients, and plotted the ROC curve of differentially expressed genes (DEGs) to analyze their diagnostic value. It was found that the AUC values of miRNA-452-5p, miRNA-5589-5p, miRNA-5589-3p, and miRNA-4661-5p in the early diagnosis of HCC were 0.830, 0.879, 0.838, and 0.725, respectively. Moreover, the four miRNAs were differentially expressed in only one or two types of cancer with good specificity, which might be used as the early diagnostic biomarkers for HCC [62]. In addition, Huang et al. found that miRNAs might also serve as the diagnostic biomarkers for lung metastasis in HCC patients, and the combination of multiple miRNAs had better performance than a single miRNA. For example, the AUC values of miRNA-18A, miRNA-20B, and miRNA-221 for the diagnosis of HCC lung metastasis were 0.7722, 0.8661, and 0.5816, respectively, while that of their combination reached 0.904, which greatly improved compared with that of a single miRNA [63]. This phenomenon was also supported by Yu et al., who analyzed and validated five GEO datasets and discovered that miRNA-184, miRNA-532-5p, miRNA-221-3p, miRNA-5589-5p, let-76-3p, and miRNA-26b-3p were significantly differentially expressed in the serum of HCC patients. Moreover, the AUC value of the diagnostic biomarker composed of these six miRNAs reached 0.9559, indicating that they had good diagnostic value [64]. Similarly, the combination of miRNAs and other diagnostic markers is also suitable for HCC. Alpha-fetoprotein (AFP) has been extensively applied in the diagnosis of various cancers, but due to its low accuracy, specificity, and sensitivity, it is not used in the diagnosis of cancer alone, but in combination with other diagnostic methods. Fouda et al. analyzed the combination of AFP with miRNAs as a diagnostic biomarker for HCC, and discovered that the AUC value of the combination of AFP with miRNA-200, miRNA-29, miRNA-21, and miRNA-355 reached 0.92 [65], indicating that combination of miRNAs with AFPs potentially enhanced the diagnostic value of HCC. 

A large number of recent studies also support miRNAs as the prognostic biomarkers of HCC. For instance, through differential analysis of HCC miRNAs in TCGA databases and stepwise multivariate Cox regression analysis of the correlation between differentially expressed miRNAs and prognosis, Zhang et al. found that miRNA-139-3p, miRNA-760, and miRNA-7-5p were correlated with the prognosis of HCC patients. Meanwhile, they detected the levels of these three miRNAs in the serum of HCC patients. As a result, compared with normal patients, the levels of miRNA-139-3p in the serum of HCC patients decreased, while those of miRNA-7-5p and miRNA-760 increased. Combined with ROC curve analysis, it was indicated that these three miRNAs were also of great diagnostic value for HCC [66]. Liver transplantation is an effective approach for the treatment of HCC, but there is always a risk of recurrence. miRNAs can be used to predict the recurrence of HCC after liver transplantation. Huang et al. screened miRNA-3200-3p and miRNA-3690 as the relevant prognostic markers for HCC recurrence after liver transplantation by analyzing HCC miRNAs in TCGA and GEO databases [67]. Yokota et al. also demonstrated that miRNA-638 served as a prognostic marker for recurrence after liver transplantation in HCC patients, and the two-year disease-free survival (DFS) rate of HCC patients in the high miRNA-638 expression group was 47.1%, while that in the low expression group was 77.4%, and patients in the high expression group were more likely to develop recurrence after surgery [68]. As discovered by Xie et al., miRNA-1269 and miRNA-421 were significantly over-expressed in tumor tissues through differential analysis of miRNA expression in tumor tissues and surrounding tissues of HCC patients, which was verified by analysis of TCGA database. Subsequent Kaplan–Meier analysis revealed that the high expression of miRNA-638 in HCC tissues was closely related to the shorter patient survival [69].

**Table 2:** MicroRNAs as biomarkers for diagnosis and prognosis of HCC.

miRNA	Sample	Expression	AUC	Biomarker	Reference
miR-452-5p	Tissue	↑	0.83	Diagnosis/Prognosis	[62]
miR-4661-5p	Tissue	↑	0.725	Diagnosis/Prognosis	[62]
miR-760	Tissue/Serum	↑	0.707	Diagnosis/Prognosis	[66]
miR-7-5p	Tissue/Serum	↑	0.641	Diagnosis/Prognosis	[66]
miR-27a	exosomes/Tissue	↑	0.8282	Diagnosis/Prognosis	[63]
miR-18a	exosomes/Tissue	↑	3-miR:0.904	Diagnosis/Prognosis	[63]
miR-20b	exosomes/Tissue	↑	3-miR:0.904	Diagnosis/Prognosis	[63]
miR-221	exosomes/Tissue	↑	3-miR:0.904	Diagnosis/Prognosis	[63]
miR-184	Serum	↑	6-miR:0.9535	Diagnosis	[64]
miR-532-5p	Serum	↑	6-miR:0.9535	Diagnosis	[64]
miR-221-3p	Serum	↑	6-miR:0.9535	Diagnosis	[64]
miR-200	Serum	↑	4-miR:0.92	Diagnosis	[65]
miR-21	Serum	↑	4-miR:0.92	Diagnosis	[65]
miR-355	Serum	↑	4-miR:0.92	Diagnosis	[65]
miR-1293	TEP miRNA	↑	0.78	Diagnosis	[70]
miR-3200-3p	Tissue	↑	/	Prognosis	[67]
miR-3690	Tissue	↑	/	Prognosis	[67]
miR-1269a	Tissue	↑	/	Prognosis	[69]
miR-421	Tissue	↑	/	Prognosis	[69]
miR-326	Tissue	↑	/	Prognosis	[71]
miR-21	Tissue	↑	/	Prognosis	[71]
miR-212-3p	Tissue	↑	/	Prognosis	[72]
miR-25	Tissue	↑	/	Prognosis	[73]
let-7e	Tissue/Cell	↑	/	Prognosis	[74]
let-7e	exosomes/Tissue	↑	/	Prognosis	[63]
miR-652	exosomes/Tissue	↑	/	Prognosis	[63]
miR-638	Cell/exosomes	↑	/	Prognosis	[68]
miR-5589-3p	Tissue	↓	0.838	Diagnosis/Prognosis	[62]
miR-5589-5p	Tissue	↓	0.879	Diagnosis/Prognosis	[62]
miR-139-3p	Tissue/Serum	↓	0.706	Diagnosis/Prognosis	[66]
miR-5589-5p	Serum	↓	6-miR:0.9535	Diagnosis	[64]
miR-26b-3p	Serum	↓	6-miR:0.9535	Diagnosis	[64]
let-7b-3p	Serum	↓	6-miR:0.9535	Diagnosis	[64]
miR-29a	Serum	↓	4-miR:0.92	Diagnosis	[65]
miR-495-3p	TEP miRNA	↓	0.76	Diagnosis	[70]
miR-125b	exosomes	↓	/	Diagnosis	[75]
miR-144/451a cluster	Tissue	↓	/	Prognosis	[76]
miR-140	Tissue	↓	/	Prognosis	[77]
mir-150-3p	Tissue/exosomes	↓	/	Prognosis	[78]

TEP: tumor-educated platelet, 3-miR: AUC values of three miRNA combinations appearing in the same paper, 4-miR: AUC values of four miRNA combinations appearing in the same paper, 6-miR: AUC values of six miRNA combinations appearing in the same paper, ↑: Up-regulated expression, ↓: Down-regulation expression.

### 3.3. Gastric Cancer

Gastric cancer (GC) is another major cause of cancer-related death, accounting for 8.2% of all cancer-related deaths. Unfortunately, due to the lack of early diagnostic markers or screening tools, many GC patients are diagnosed as the advanced stage, resulting in the low (3%) 5-year survival rate [79,80]. The sensitivity and specificity of existing biomarkers for GC diagnosis and prognosis are low. Currently, GC is diagnosed based on invasive procedures such as upper gastrointestinal endoscopy [81]. Some studies have shown that the abnormal expression of miRNA in GC may have clinical utility, especially in the diagnosis and prognosis of GC. miRNAs associated with GC identified in 2021 are summarized in Table 3.

Identifying effective biomarkers for the early diagnosis of GC is an effective way to improve the survival rate of GC patients, and ROC curve is an effective indicator for judging the diagnostic effect. Chen et al. analyzed the diagnostic effect of miRNA-130b by drawing ROC curve. According to their results, the AUC value of miRNA-130b was 0.911, which might serve as the potential GC diagnostic marker for further research [79]. The combination of miRNAs can also be used for the early diagnosis of GC, which achieves better diagnostic effect than a single miRNA. For example, Kohrboa et al. identified four significantly up-regulated miRNAs in the serum exosomes of GC patients, namely, miRNA-10a-5p, miRNA-19b-3p, miRNA-215-5p, and miRNA-18a-5p. ROC curve analysis showed that the AUC values were 0.801, 0.721, 0.780, and 0.736, respectively, which were lower than that of 0.813 for the combination of these four miRNAs [82]. Yu et al. selected six differentially expressed miRNAs in tumor tissues and plasma of GC patients, among which, miRNA-3185, miRNA-6083, miRNA-6792-3p, and miRNA-659-3p were up-regulated, whereas miRNA-936 and miRNA-1306-3p were down-regulated. Further analysis of their diagnostic value showed that the AUC values of the four up-regulated miRNAs were 0.825, significantly higher than those of the two down-regulated miRNAs of 0.730 [83]. In addition, in some studies, the combination of miRNAs with tumor markers currently used in the clinical diagnosis of GC also improves the diagnostic effect. For example, So et al. analyzed 578 miRNAs in the serum of 682 GC patients and identified 12 miRNAs for GC diagnosis, with an AUC value of 0.848. However, the AUC value increased to 0.884 if the 12 miRNAs were combined with age, CEA, and Helicobacter pylori serological examination [84].

Similar to diagnosis, the combination of multiple miRNAs as a prognostic marker is definitely better than a single miRNA, so the researchers set out to construct a prognostic model including multiple miRNAs. Xu et al. obtained nine immune-related miRNAs through TCGA database, and verified that these nine miRNAs were closely related to GC prognosis. Among them, six miRNAs were used as the GC-related tumor suppressors, and their high expression was positively correlated with longer patient survival, whereas the other three miRNAs were high-risk miRNAs, and their high expression predicted poor prognosis. Thereafter, the authors constructed the immune related signature (ImmiRSig) based on these nine miRNAs, and divided the patients into high-risk group and low-risk group. According to their results, the expression of high-risk miRNAs in low-risk patients was lower than that in high-risk patients, while that of protective miRNAs showed an opposite trend [85]. Similarly, Liu et al. also constructed the GI-related miRNAs signature (GIMiSig) for predicting the survival rate of GC patients based on eight miRNAs, and compared it with the current clinically commonly used cancer staging methods. As a result, GIMiSig achieved a better prediction effect on the survival rate of GC patients [86]. In addition, Qi et al. built a risk scoring formula based on three miRNAs including miRNA-126-3p, miRNA-143-5p, and miRNA-1275, which was verified and might be used to predict the prognosis of GC patients [87].

**Table 3:** MicroRNAs as biomarkers for diagnosis and prognosis of GC.

miRNA	Sample	Expression	AUC	Biomarker	Reference
miR-455-3p	Tissue	↑	3-miR:0.91	Diagnosis	[88]
miR-135b-5p	Tissue	↑	3-miR:0.91	Diagnosis	[88]
let-7a-3p	Tissue	↑	3-miR:0.91	Diagnosis	[88]
miR-3185	Tissue/Plasma	↑	4-miR:0.825	Diagnosis	[83]
miR-6083	Tissue/Plasma	↑	4-miR:0.825	Diagnosis	[83]
miR-6792-3p	Tissue/Plasma	↑	4-miR:0.825	Diagnosis	[83]
miR-659-3p	Tissue/Plasma	↑	4-miR:0.825	Diagnosis	[83]
miR-140	Serum	↑	12-miR:0.848	Diagnosis	[84]
miR-183	Serum	↑	12-miR:0.848	Diagnosis	[84]
miR-30e	Serum	↑	12-miR:0.848	Diagnosis	[84]
miR-103a	Serum	↑	12-miR:0.848	Diagnosis	[84]
miR-93	Serum	↑	12-miR:0.848	Diagnosis	[84]
miR-142	Serum	↑	12-miR:0.848	Diagnosis	[84]
miR-21	Serum	↑	12-miR:0.848	Diagnosis	[84]
miR-29c	Serum	↑	12-miR:0.848	Diagnosis	[84]
miR-424	Serum	↑	12-miR:0.848	Diagnosis	[84]
miR-340	Serum	↑	12-miR:0.848	Diagnosis	[84]
miR-1290	Serum	↑	0.6576	Diagnosis	[89]
miR-320a	Serum	↑	≥0.95	Diagnosis	[90]
miR-1260b	Serum	↑	≥0.95	Diagnosis	[90]
miR-6515-5p	Serum	↑	≥0.95	Diagnosis	[90]
miR-130b	Plasma	↑	0.911	Diagnosis	[79]
miR-10a-5p	exosomes	↑	0.801	Diagnosis	[82]
miR-19b-3p	exosomes	↑	0.721	Diagnosis	[82]
miR-215-5p	exosomes	↑	0.78	Diagnosis	[82]
miR-18a-5p	exosomes	↑	0.736	Diagnosis	[82]
miR-145-3p	Tissue	↑	/	Prognosis	[85]
miR-328-3p	Tissue	↑	/	Prognosis	[85]
miR-125b-5p	Tissue	↑	/	Prognosis	[85]
miR-99a-3p	Tissue	↑	/	Prognosis	[85]
miR-133a-5p	Tissue	↑	/	Prognosis	[85]
miR-1292-5p	Tissue	↑	/	Prognosis	[85]
miR-196b-3p	Tissue	↑	/	Prognosis	[86]
miR-548v	Tissue	↑	/	Prognosis	[86]
miR-380-3p	Tissue	↑	/	Prognosis	[86]
miR-1275	Tissue	↑	/	Prognosis	[86]
miR126-3p	Tissue	↑	/	Prognosis	[87]
miR-143-5p	Tissue	↑	/	Prognosis	[87]
miR-424-5p	Tissue	↑	/	Prognosis	[91]
let-7a-5p	Tissue	↑	/	Prognosis	[91]
miR-27a-3p	Tissue	↑	/	Prognosis	[91]
miR-126-5p	Tissue	↑	/	Prognosis	[91]
miR-182	/	↑	/	Prognosis	[92]
miR-204-5p	Tissue	↓	4-miR:0.94	Diagnosis	[88]
miR-149-5p	Tissue	↓	4-miR:0.94	Diagnosis	[88]
miR-143-3p	Tissue	↓	4-miR:0.94	Diagnosis	[88]
miR-195-5p	Tissue	↓	4-miR:0.94	Diagnosis	[88]
miR-936	Tissue/Plasma	↓	2-miR:0.73	Diagnosis	[83]
miR-1306-3p	Tissue/Plasma	↓	2-miR:0.73	Diagnosis	[83]
miR-181a	Serum	↓	12-miR:0.848	Diagnosis	[84]
miR-126	Serum	↓	12-miR:0.848	Diagnosis	[84]
miR-1343-3p	Serum	↓	1.000	Diagnosis	[93]
miR-590-5p	exosomes	↓	0.81	Diagnosis/Prognosis	[94]
miR-92b-5p	Tissue	↓	/	Prognosis	[85]
miR-942-3p	Tissue	↓	/	Prognosis	[85]
miR-675-3p	Tissue	↓	/	Prognosis	[85]
miR-125b-5p	Tissue	↓	/	Prognosis	[86]
miR-99a-3p	Tissue	↓	/	Prognosis	[86]
miR-100-5p	Tissue	↓	/	Prognosis	[86]
miR-363-3p	Tissue	↓	/	Prognosis	[86]
miR-1275	Tissue	↓	/	Prognosis	[87]
miR-23b	Tissue	↓	/	Prognosis	[95]
miR-194	Tissue/Cell	↓	/	Prognosis	[96]
miR-339	Tissue/Cell	↓	/	Prognosis	[97]
miR-148a	Plasma	↓	/	Prognosis	[98]

4-miR: AUC values of four miRNA combinations appearing in the same paper, 2-miR: AUC values of two miRNA combinations appearing in the same paper, 12-miR: AUC values of twelve miRNA combinations appearing in the same paper, 3-miR: AUC values of three miRNA combinations appearing in the same paper, ↑: Up-regulated expression, ↓: Down-regulation expression.

### 3.4. Colorectal Cancer

Colorectal cancer (CRC) is a common malignant tumor; however, the effective diagnostic and prognostic markers are lacking, and the time of diagnosis will have a significant impact on the patient survival rate. Relevant studies have suggested that the survival rate of stage I CRC is 93%, but it drops sharply to 8% in stage IV CRC [99]. At present, colonoscopy is the gold standard for CRC diagnosis, but it is highly invasive and cumbersome. In addition, fecal occult blood test is also one of the commonly used diagnostic methods for CRC, but it has low sensitivity and specificity [100]. miRNAs are frequently dysregulated in CRC and used as novel and promising biomarkers because they have less invasibility, but good sensitivity and specificity. The miRNAs associated with CRC discovered in 2021 are summarized in Table 4.

Some miRNAs such as miRNA-1290 may have diagnostic value in different tumors, which can be used as the diagnostic markers not only for GC but also for CRC [89]. Silva et al. constructed a signature by combining four miRNAs (let-7e-5p, miRNA-106a-5p, miRNA-28-3p, miRNA-542-5p), which not only distinguished CRC patients from healthy subjects, but also from thickened polyps and adenomas [101]. Meanwhile, miRNAs can also be used to distinguish metastatic from non-metastatic CRC patients. Chen et al. analyzed miRNAs in plasma of CRC patients with liver metastasis, CRC patients without liver metastasis, and healthy subjects. According to their results, the AUC value of miRNA-96 combined with miRNA-99B in CRC patients and healthy subjects was 0.93, and that in patients with liver metastasis was 0.91 [102]. Nassar et al. found that miRNA-210 and miRNA-203 also showed certain diagnostic value in CRC liver metastasis [103]. In addition to its diagnostic role in liver metastasis, miRNAs can also be used in lymph node metastasis of CRC. Dokhanchi et al. discovered a correlation between miRNA-19A-3p, miRNA-203-3p, and miRNA-221-3p expression and lymph node metastasis in serum of CRC patients [104]. 

Some miRNAs play different roles in CRC, which not only have diagnostic value, but can also be used as prognostic markers for CRC patients. Similarly, miRNA-96 and miRNA-99b have such functions, which are thereby included in the nomogram prognostic model composed of five independent prognostic factors constructed by Chen et al. [102]. In addition, Liu et al. identified and verified that miRNA-216-5p, miRNA-194-3p, and miRNA-3677-3p, which were included in the construction of immune correlation miRNAs prognostic signature (IAMIPS) module, not only predicted the survival of CRC patients well, but also predicted the sensitivity of chemotherapeutic drugs [105]. Lan et al. analyzed autophagy-related miRNAs in tumor tissues of CRC patients and found that low expression of miRNA-449 was associated with autophagy and poor overall survival (OS) [106]. In addition, other miRNAs are studied in CRC-related cell lines. For example, Cho et al. indicated that miRNA-193a and let-7g were significantly differentially expressed between CRC cell lines and peritoneal metastasis (PTM) cell lines. Moreover, they were associated with poorer prognosis (including recurrence, venous invasion, and lymphatic invasion) in plasma exosomes from CRC patients [107]. In most studies, people only focus on miRNAs expression at a certain time point, but ignore the changes of miRNAs in the whole course of disease, as a result, individual differences between patients will affect the results. Fukada et al. prospectively focused on the changes of miRNA-21-5p throughout the disease course of CRC patients, including before surgery, 7 days, 1 month, and 6 months after surgery. According to their results, miRNA-21-5p levels were significantly higher in plasma of CRC patients at recurrence, 1 and 6 months after surgery [108], greatly improving the accuracy of the marker.

**Table 4:** MicroRNAs as biomarkers for diagnosis and prognosis of CRC.

miRNA	Sample	Expression	AUC	Biomarker	Reference
miR-1290	Serum	↑	0.7852	Diagnosis	[89]
miR-19a-3p	Serum	↑	0.84	Diagnosis	[104]
miR-203-3p	Serum	↑	0.83	Diagnosis	[104]
miR-221-3p	Serum	↑	0.88	Diagnosis	[104]
let-7f-5p	Serum	↑	0.73	Diagnosis	[104]
miR-21	Serum/Plasma	↑	0.87	Diagnosis	[109]
let-7e-5p	Plasma/Tissue	↑	4-miR:	Diagnosis	[101]
miR-106a-5p	Plasma/Tissue	↑	0.716(Plasma);	Diagnosis	[101]
miR-28-3p	Plasma/Tissue	↑	0.998(Tissue)	Diagnosis	[101]
miR-542-5p	Plasma/Tissue	↑		Diagnosis	[101]
miR-210	Plasma	↑	2-miR:0.731	Diagnosis	[103]
miR-21	Plasma	↑	2-miR:0.731	Diagnosis	[103]
miR-203	Plasma	↑	2-miR:0.833	Diagnosis	[103]
miR-210	Plasma	↑	2-miR:0.833	Diagnosis	[103]
miR-15b	exosomes	↑	0.82	Diagnosis	[100]
miR-16	exosomes	↑	0.58	Diagnosis	[100]
miR-21	exosomes	↑	0.75	Diagnosis	[100]
miR-31	exosomes	↑	0.75	Diagnosis	[100]
miR-183	Tissue	↑	/	Prognosis	[99]
miR-20a	Tissue	↑	/	Prognosis	[99]
miR-21	Tissue	↑	/	Prognosis	[99]
miR-216a-5p	Tissue	↑	/	Prognosis	[105]
let-7c	Tissue	↑	/	Prognosis	[110]
let-7e	Tissue	↑	/	Prognosis	[110]
miR-34c	Tissue	↑	/	Prognosis	[110]
miR-133b	Tissue	↑	/	Prognosis	[110]
miR-21-5p	Plasma	↑	/	Prognosis	[108]
let-7g	exosomes	↑	/	Prognosis	[107]
miR-96	Plasma	↑	2-miR:0.93	Diagnosis/Prognosis	[102]
miR-99b	Plasma	↓	2-miR:0.93	Diagnosis/Prognosis	[102]
miR-381-3p	exosomes	↓	2-miR:0.807	Diagnosis	[111]
miR-377-3p	exosomes	↓	2-miR:0.807	Diagnosis	[111]
miR-195	Tissue	↓	/	Prognosis	[99]
miRN-139	Tissue	↓	/	Prognosis	[99]
miR-145	Tissue	↓	/	Prognosis	[99]
miR-194-3p	Tissue	↓	/	Prognosis	[105]
miR-3677-3p	Tissue	↓	/	Prognosis	[105]
miR-449a	Tissue	↓	/	Prognosis	[106]
miR-106a	Tissue	↓	/	Prognosis	[110]
miR-144	Tissue	↓	/	Prognosis	[110]
miR-100	Tissue	↓	/	Prognosis	[112]
miR-99a	Tissue	↓	/	Prognosis	[112]
miR-33b-5p	Tissue	↓	/	Prognosis	[113]
miR-193a	exosomes	↓	/	Prognosis	[107]

2-miR: AUC values of two miRNA combinations appearing in the same paper, 4-miR: AUC values of four miRNA combinations appearing in the same paper, ↑: Up-regulated expression, ↓: Down-regulation expression.

### 3.5. Breast Cancer

According to the International Agency for Research on Cancer (IARC) managed by the World Health Organization (WHO), breast cancer (BC) currently accounts for the largest number of cancer cases and deaths in women. Early diagnosis of BC is one of the important strategies to reduce BC-related mortality. Numerous studies have reported that the 5-year survival rate of stage I BC patients can reach 100%, but less than 50% of BC patients are diagnosed in the early stage [114,115]. There is growing evidence that early diagnosis holds the key toward effective treatment outcome. Thus, multiple studies have concentrated on exploring biomarkers for BC detection. BC-related miRNAs discovered in 2021 are summarized in Table 5.

The early and combined diagnosis of miRNAs has an important guiding role in the treatment of BC patients. For example, Anna et al. analyzed miRNAs in plasma of 54 BC patients and found that the combination of miRNA-30b-5p with miRNA-99a-5p showed good diagnostic value for early BC patients, and its AUC value was 0.9273. Such results were validated in the plasma of another 74 BC patients (AUC: 0.7620) [116]. Itani et al. also selected four miRNAs, miRNA-145, miRNA-139-5p, miRNA-130a, and miRNA-425-5p, as the diagnostic markers for early BC patients, and its diagnostic performance was superior to any of the two miRNAs combinations, with an AUC value of 0.97 [117]. Apart from mature miRNAs, pri-miRNA and exosomal miRNAs also served as the important markers for BC diagnosis. Majumder et al. analyzed the initial transcript of miRNAs, according to their results, pri-miRNA-526b was a diagnostic marker for distinguishing stage I BC patients from healthy controls, and the AUC value was 0.7273 [118]. In addition, Kim et al. analyzed the diagnostic power of miRNAs for each subtype of BC in more detail. By analyzing the data from TCGA database and verifying the expression of miRNAs in extracellular vesicles of BC cell line and plasma of BC patient, they obtained four up-regulated miRNAs, namely miRNA-16, miRNA-21, miRNA-9, and miRNA-429. Further studies revealed that these miRNAs not only served as diagnostic markers for BC separately, but could also be used as a combination for the diagnosis of BC with better results [119]. In addition to the combination of multiple miRNAs, some researchers have turned their attention to miRNA clusters. For instance, Lal et al. determined the diagnostic value of miRNA379/656 cluster (the second largest gene cluster in humans, containing 42 miRNA-encoding genes) in BC. As a result, 15 miRNAs were selected as a subset of miRNA379/656, with an AUC value of 0.9843 [120].

Prognosis is an important indicator for the detection of BC recurrence and therapeutic effect, and miRNAs clusters and combinations can also be used as prognostic markers for BC. For example, Lal et al. found that low expression of the miRNA379/656 cluster was associated with poor clinical outcomes in patients [120]. In addition, Tian et al. identified a novel 5-miRNAs (including miRNA-574, miRNA-224, miRNA-210, miRNA-30b, miRNA-130a) prognostic model, which was further used to construct a nomogram by combining the prognostic model with traditional clinical prognostic predictors (like age, tumor size, lymph node metastasis, and subtype) for the better application in clinical settings [121]. Turkistani et al. directly screened three groups of miRNAs combinations, which were respectively used to determine the tumor size (≥5 cm or <5 cm), lymph node metastasis and recurrence of BC patients, so as to better understand the causes of poor prognosis [122] and to improve patient prognosis in a targeted clinical application. In addition to tumor size, lymph node metastasis, and recurrence, resistance to chemotherapeutics was another important cause of the poor prognosis of BC patients. Ai et al. directly focused more on miRNAs related to chemotherapy resistance, by comparing the differences in miRNAs expression profiles in tissue samples between chemo-sensitive and chemo-resistant patients. According to their results, the combination of miRNA-200C-3p, miRNA-214-3p, miRNA-23A-3p, miRNA-451A, and miRNA-638 was applicable for the prediction of chemotherapy resistance in BC patients [123]. Apart from miRNAs, the ncRNAs axis is also an important indicator for future BC prognosis. For instance, Lin et al. found the lncRNA GATA3-AS1/miRNA-495-3p/CENPU axis with high prognostic value in BC tissues by searching and analyzing multiple ncRNAs databases [124], indicating the increasing diversity of BC prognostic biomarkers.

**Table 5:** MicroRNAs as biomarkers for diagnosis and prognosis of BC.

miRNA	Sample	Expression	AUC	Biomarker	Reference
let-7b-5p	Serum/Tissue/exosomes	↑	>0.94	Diagnosis	[125]
miR-106a-5p	Serum/Tissue/exosomes	↑	>0.94	Diagnosis	[125]
miR-19a-3p	Serum/Tissue/exosomes	↑	>0.94	Diagnosis	[125]
miR-19b-3p	Serum/Tissue/exosomes	↑	>0.94	Diagnosis	[125]
miR-20a-5p	Serum/Tissue/exosomes	↑	>0.94	Diagnosis	[125]
miR-223-3p	Serum/Tissue/exosomes	↑	>0.94	Diagnosis	[125]
miR-25-3p	Serum/Tissue/exosomes	↑	>0.94	Diagnosis	[125]
miR-425-5p	Serum/Tissue/exosomes	↑	>0.94	Diagnosis	[125]
miR-451a	Serum/Tissue/exosomes	↑	>0.94	Diagnosis	[125]
miR-92a-3p	Serum/Tissue/exosomes	↑	>0.94	Diagnosis	[125]
miR-93-5p	Serum/Tissue/exosomes	↑	>0.94	Diagnosis	[125]
miR-16-5p	Serum/Tissue/exosomes	↑	>0.94	Diagnosis	[125]
miR-451a	Serum	↑	6-miR:0.881	Diagnosis	[126]
miR-195-5p	Serum	↑	6-miR:0.881	Diagnosis	[126]
miR-126-5p	Serum	↑	6-miR:0.881	Diagnosis	[126]
miR-30b-5p	Plasma	↑	2-miR:0.9273	Diagnosis	[116]
miR-99a-5p	Plasma	↑	2-miR:0.9273	Diagnosis	[116]
miR-145	Plasma	↑	4-miR:0.97	Diagnosis	[117]
miR-139-5p	Plasma	↑	4-miR:0.97	Diagnosis	[117]
miR-130a	Plasma	↑	4-miR:0.97	Diagnosis	[117]
miR-425-5p	Plasma	↑	4-miR:0.97	Diagnosis	[117]
pri-miR-526b	Plasma	↑	0.7273	Diagnosis	[118]
miR-19a	Plasma	↑	4-miR:0.802	Diagnosis	[127]
miR-20a	Plasma	↑	4-miR:0.802	Diagnosis	[127]
miR-421	exosomes	↑	0.835	Diagnosis	[128]
miR-128	exosomes	↑	0.825	Diagnosis	[128]
miR-16	EV	↑	4-miR:0.88	Diagnosis	[119]
miR-21	EV	↑	4-miR:0.88	Diagnosis	[119]
miR-9	EV	↑	4-miR:0.88	Diagnosis	[119]
miR-429	EV	↑	4-miR:0.88	Diagnosis	[119]
miR-10b	Tissue	↑	/	Prognosis	[53]
miR-210	Tissue	↑	/	Prognosis	[121]
miR-378c	Tissue	↑	/	Prognosis	[122]
let-7f-5p	Tissue	↑	/	Prognosis	[122]
miR-1268b	Tissue	↑	/	Prognosis	[122]
miR-200c-3p	Tissue	↑	/	Prognosis	[122]
miR-1271-3p	Tissue	↑	/	Prognosis	[122]
miR-200c-3p	Tissue	↑	/	Prognosis	[123]
miR-214-3p	Tissue	↑	/	Prognosis	[123]
miR-23a-3p	Tissue	↑	/	Prognosis	[123]
miR-106b-5p	Tissue	↑	/	Prognosis	[128]
miR-93	Serum	↑	/	Prognosis	[129]
miR-210	Serum	↑	/	Prognosis	[129]
miR-19a	Serum	↑	/	Prognosis	[129]
miR-19b	Serum	↑	/	Prognosis	[129]
miR379/656 cluster	Tissue	↓	0.9843	Diagnosis/ Prognosis	[120]
miR-423-3p	Serum	↓	6-miR:0.881	Diagnosis	[126]
miR-192-5p	Serum	↓	6-miR:0.881	Diagnosis	[126]
miR-17-5p	Serum	↓	6-miR:0.881	Diagnosis	[126]
miR-126	Plasma	↓	4-miR:0.802	Diagnosis	[127]
miR-155	Plasma	↓	4-miR:0.802	Diagnosis	[127]
miR-203	Tissue	↓	/	Prognosis	[53]
miR-574	Tissue	↓	/	Prognosis	[121]
miR-224	Tissue	↓	/	Prognosis	[121]
miR-30b	Tissue	↓	/	Prognosis	[121]
miR-130a	Tissue	↓	/	Prognosis	[121]
miR-1200	Tissue	↓	/	Prognosis	[122]
miR-1249-3p	Tissue	↓	/	Prognosis	[122]
miR-1255b-5p	Tissue	↓	/	Prognosis	[122]
miR-520d	Tissue	↓	/	Prognosis	[122]
miR-527	Tissue	↓	/	Prognosis	[122]
miR-518a-5p	Tissue	↓	/	Prognosis	[122]
miR-2117	Tissue	↓	/	Prognosis	[122]
miR-638	Tissue	↓	/	Prognosis	[123]
miR-451a	Tissue	↓	/	Prognosis	[123]
miR-495-3p	Tissue	↓	/	Prognosis	[124]
miR-223	Tissue	↓	/	Prognosis	[130]
miR-34a	Tissue	↓	/	Prognosis	[131]
miR-200c	Tissue	↓	/	Prognosis	[131]
miR-363-5p	Tissue/exosomes	↓	/	Prognosis	[132]

EV: extracellular vesicles, 4-miR: AUC values of four miRNA combinations appearing in the same paper, 2-miR: AUC values of two miRNA combinations appearing in the same paper, 6-miR: AUC values of six miRNA combinations appearing in the same paper, ↑: Up-regulated expression, ↓: Down-regulation expression.

### 3.6. Prostate Cancer

Prostate cancer (PCa) is the most common and leading cause of death in men. At present, the common methods for clinical diagnosis of PCa include tissue biopsy, digital rectal examination, and serum prostate specific antigen (PSA). Tissue biopsy and digital rectal examination are highly invasive, while the low specificity of PSA has led to missed cases of the prostate-related diseases including PCa and benign prostatic hyperplasia (BPH), thereby limiting its clinical application in PCa [133,134]. These traditional assays do not meet the current needs, so a variety of new methods have been developed with special attention to sensitivity, specificity, and invasiveness. The PCa-related miRNAs identified in 2021 are summarized in Table 6.

Accurate diagnosis provides a basic guarantee for the treatment of PCa, and serum PSA is an important indicator for the determination of PCa. When the PSA concentration is 4–10 ng/mL, it is difficult to distinguish PCa from BPH, so other biomarkers are necessary to achieve the accurate diagnosis of PCa. MiRNAs are the most commonly used auxiliary markers, especially miRNAs in serum and urine, which are characterized by their invasiveness and less damage to patients. For example, Mello-Grand et al. analyzed 102 plasma samples with the PSA concentration of 4–16 ng/mL (53 PCa-negative and 49 PCa-positive). According to their results, the up-regulated miRNA-5100 helped to distinguish PCa from BPH [135]. In addition, Giglio et al. analyzed miRNAs in the plasma of 290 PCa patients, and the screened combination of miRNA-26b-5p and miRNA-98-5p performed well in distinguishing PCa from BPH patients (AUC of 0.944). Meanwhile, another combination of miRNA-26b-5p and miRNA-4732-3p also exhibited favorable effect (AUC of 0.80) on diagnosing advanced and early PCa [136]. Urine miRNAs can also be used to differentiate PCa from BPH. Compared with serum and tissues, urine samples can be obtained without any harm to patients. Markert et al. obtained six miRNAs (miRNA-15a-5p, miRNA-3126-3p, miRNA-324-5p, miRNA-150-5p, miRNA-425-3p, miRNA-6078) by analyzing the miRNAs in the urine of PCa and BPH patients. As a result, these miRNAs could be used to distinguish PCa from BPH, and their AUC values were all ≥0.70 [137]. Metastasis is another key factor affecting the survival of patients with PCa, and bone metastasis plays a dominant role. Wang et al. found that exosomes miRNA-181A-5p served as a biomarker for the early diagnosis of PCa bone metastasis in the serum of PCa patients [138]. At present, one of the treatments for metastatic PCa is androgen-deprivation therapy (ADT), but many patients develop resistance to ADT and progressed into ADT-resistant prostate cancer (CRPC). By comparing the differentially expressed miRNAs in tumor tissues between CRPC and primary PCa patients, Ronnau et al. obtained the combination of three miRNAs (miRNA-205, miRNA-3195 and miRNA-4417), which could be used as a potential biomarker for differentiating CRPC from primary PCa [139]. The abnormal expression of miRNAs in the tissues of PCa patients can be used to determine patient prognosis as well, and the prognosis is directly related to the clinical therapeutic effect and metastasis of PCa. Stoen et al. discovered that the high expression of miRNA-17-5p in tumor epithelium was associated with biochemical disorder (BF) and clinical failure (CF) in PCa patients, which was one of the factors contributing to the poor prognosis of PCa patients [140]. Additionally, Liu et al. indicated that the expression of miRNA-199b-3p in PCa was significantly lower than that in BPH by analyzing the expression of miRNAs in tumor tissues from 60 PCa and BPH patients. The low expression of miRNA-199b-3p was positively correlated with poor progression-free survival (PFS) in PCa patients [141]. Combinations of multiple miRNAs can be used to predict the prognosis of PCa patients. Bian et al. obtained the combination of 15 miRNAs by TCGA databases, which was adopted to predict the relapse-free survival (RFS) rate of PCa patients [142]. Similar to diagnosis, urinary miRNAs served as the prognostic markers for PCa. Kim et al. discovered that exosomal miRNA-532-5p was significantly up-regulated in urine of biochemical recurrence (BRC) patients compared with non-BRC patients, which was used as a marker to predict BRC in PCa patients after radical prostatectomy (RP) by KM analysis [143].

**Table 6:** MicroRNAs as biomarkers for diagnosis and prognosis of PCa.

miRNA	Sample	Expression	AUC	Biomarker	Reference
miR-3195	Tissue	↑	3-miR:0.994	Diagnosis	[139]
miR-4417	Tissue	↑	3-miR:0.994	Diagnosis	[139]
miR-301a	Serum/Tissue	↑	/	Diagnosis	[144]
miR-4732-3p	Plasma	↑	2-miR:0.8	Diagnosis	[136]
miR-5100	Plasma	↑	/	Diagnosis	[135]
miR-1255-5p	Plasma	↑	0.885	Diagnosis	[145]
miR-181a-5p	exosomes	↑	0.738	Diagnosis	[138]
miR-451a	Urine exosomes	↑	0.757	Diagnosis	[146]
miR-486-3p	Urine exosomes	↑	0.704	Diagnosis	[146]
miR-486-5p	Urine exosomes	↑	0.796	Diagnosis	[146]
miR-6078	Urine	↑	0.7	Diagnosis	[137]
miR-1913	Urine	↑	2-miR:0.821	Diagnosis	[147]
miR-3659	Urine	↑	2-miR:0.821	Diagnosis	[147]
miR-451a	SEVs	↑	0.65	Diagnosis	[148]
miR-141	SEVs	↑	0.64	Diagnosis	[148]
miR-145	SEVs	↑	0.76	Diagnosis	[148]
miR-221	SEVs	↑	0.7	Diagnosis	[148]
miR-17-5p	Tissue	↑	/	Prognosis	[140]
miR-222-3p	Tissue	↑	/	Prognosis	[142]
miR-582-5p	Tissue	↑	/	Prognosis	[142]
miR-582-3p	Tissue	↑	/	Prognosis	[142]
miR-505-3p	Tissue	↑	/	Prognosis	[142]
miR-326	Tissue	↑	/	Prognosis	[142]
miR-212-3p	Tissue	↑	/	Prognosis	[142]
miR-296-5p	Tissue	↑	/	Prognosis	[142]
miR-144-3p	Tissue	↑	/	Prognosis	[142]
miR-532-5p	Urine exosomes	↑	/	Prognosis	[143]
miR-425-5p	Cell exosomes	↑	/	Prognosis	[149]
miR-205	Tissue	↓	3-miR:0.994	Diagnosis	[139]
miR-26b-5p	Plasma	↓	2-miR:0.944	Diagnosis	[136]
miR-98-5p	Plasma	↓	2-miR:0.944	Diagnosis	[136]
miR-26b-5p	Plasma	↓	2-miR:0.8	Diagnosis	[136]
miR-15a-5p	Urine	↓	0.71	Diagnosis	[137]
miR-3126-3p	Urine	↓	0.76	Diagnosis	[137]
miR-324-5p	Urine	↓	0.74	Diagnosis	[137]
miR-150-5p	Urine	↓	0.76	Diagnosis	[137]
miR-425-3p	Urine	↓	0.71	Diagnosis	[137]
miR-375	Urine exosomes	↓	0.788	Diagnosis	[146]
miR-199b-3p	Tissue	↓	/	Prognosis	[141]
miR-21-5p	Tissue	↓	/	Prognosis	[142]
miR-192-5p	Tissue	↓	/	Prognosis	[142]
miR-15b-5p	Tissue	↓	/	Prognosis	[142]
miR-106b-5p	Tissue	↓	/	Prognosis	[142]
miR181a-5p	Tissue	↓	/	Prognosis	[142]
miR-18a-5p	Tissue	↓	/	Prognosis	[142]
miR-301a-3p	Tissue	↓	/	Prognosis	[142]

SEVs: small extracellular vesicles, 2-miR: AUC values of two miRNA combinations appearing in the same paper, 3-miR: AUC values of three miRNA combinations appearing in the same paper, ↑: Up-regulated expression, ↓: Down-regulation expression.

## 4. Conclusions and Prospects

At present, there are numerous studies about miRNAs and cancer diagnosis and prognosis, but few results have been actually applied in the clinic. However, there are some miRNAs products used to detect tumor patient samples, such as miRNAs detection kits, test strips, and sensors. For instance, Zhou et al. developed a dual colorimetric miRNAs detection kit based on constant-temperature PCR technology, which rapidly and qualitatively detected miRNA-223 and miRNA-200b at room temperature, and was used to distinguish lung cancer patients from healthy people [150]. Yu et al. developed a novel surface-enhanced Raman scattering (SERS)-lateral flow assay (LFA) strip that simultaneously measured miRNA-21 and miRNA-196a-5p on a single test line in urine samples of NSCLC patients, which improved the detection efficiency and was adopted for the diagnosis of NSCLC [151]. Additionally, Zhuang et al. developed electrochemical biosensors for the detection of miRNA-100 in serum of GC patients [152], and Li et al. developed a sensor for the detection of miRNA-21 in serum exosomes of BC patients [153]. In China, some miRNAs detection kits have completed product registration and can thereby be used in clinical practice, including 7 miRNA detection kits (Jusbio, China), miR-92a detection kits (GeneBiohealth, China), and miRNA-25 detection kit (MicroMedMark, China), which can be utilized for the diagnosis and efficacy monitoring of liver cancer, colon cancer and pancreatic cancer, respectively.

Ideally, miRNAs may be more effective than the methods that have been applied in clinical diagnosis and prognosis, but there are still some problems in practical research. For instance, (1) small sample size: in the literature retrieved in this work, the maximum sample size is 682 serum samples from GC patients [84], whereas the smaller sample size may be only a few cases, and some are completely based on databases such as TCGA and GEO. (2) The sampling method was highly invasive to patients: many studies used tissue biopsy samples instead of blood, urine, and other samples that were less harmful to patients. (3) A single miRNA was used as a marker for the diagnosis and prognosis of some diseases: It was obvious that a combination of multiple miRNAs or a combination of miRNAs with clinically applied markers would be better than a single miRNA. (4) Biomarker detection at a single time point of the disease: in many studies, researchers only focused on miRNAs expression at a certain time point of the disease, but ignored the changes of miRNAs during the whole course of disease, resulting in the deviation of results due to individual differences of patients. (5) Tumor heterogeneity of miRNAs: the expression and functions of some miRNAs were different in different cancers, if the miRNA screened from one cancer directly applied to the clinic, it may interfere with the detection results of other diseases, and their clinical application will be limited. Therefore, miRNAs as biomarkers for disease diagnosis and prognosis can be appropriately improved in subsequent studies. For example, the relevant medical staff should be trained regularly, so that they can understand the detailed information of a miRNA as a marker of disease diagnosis and prognosis as comprehensively as possible, so that the accuracy of detection can be greatly improved. First, as many patient samples as possible should be collected. Second, less invasive sample types such as blood and urine should be used. Third, many combinations of miRNAs, miRNAs, and other ncRNAs, and miRNAs and biochemical markers should be used to improve disease diagnosis and prognosis. Fourth, changes in miRNAs expression during the entire course of disease should be explored as far as possible to eliminate the influence of individual differences on screening results. Fifth, the heterogeneity of miRNAs in diseases should be fully understood and verified as far as possible to reduce the impact on the detection results of other diseases.

Certainly, miRNAs also have as many advantages as tumor biomarkers. For example, miRNAs are stable and can be easily detected in different tissues, blood, urine, stool, saliva, and ascites. There are various sample types, especially blood and urine samples, which are less invasive to patients and easy to be accepted by patients. Furthermore, their accuracy as diagnostic or prognostic markers is higher than AFP, CEA, PSA, and other biochemical markers that have been applied in clinical practice. Therefore, in the near future, miRNA will be studied more widely. With the further improvement of biomarker research system, as well as the development of bioinformatics and diagnostic technology, more miRNAs will be gradually applied in the clinical diagnosis and prognosis of cancer.

## Data Availability

Not applicable.

## Abbreviations

miRNAs(miR)	MicroRNAs
ncRNAs	non-coding RNAs
IARC	International Agency for Research on Cancer
NCC	National Cancer Center of China
CT	Computed Tomography
MRI	magnetic resonance
PET	Positron emission computed tomography
CEA	carcinoembryonic antigen
AFP	alpha-fetoprotein
PSA	Prostate cancer specific antigen
CTC	circulating tumor cells
NSCLC	non-small cell lung cancer
RISC	RNA induced silencing complex
pri-miRNA	primary miRNA
pre-miRNA	precursor miRNA
UTR	untranslated region
PTGES3	prostaglandin E synthase 3
FAP	ibroblast activation protein
PD1	programmed cell death 1
PDL1	programmed death ligand 1
LUAD	lung adenocarcinoma
LUSC	lung squamous cell carcinoma
CSF	cerebrospinal fluid
ROC	receiver operating characteristic
AUC	area under the curve
PTEN	phosphatase and tensin homolog
HCC	hepatocellular carcinoma
DFS	disease-free survival
GC	Gastric cancer
ImmiRSig	immune-related nine-miRNA signature
GIMiSig	GI-related miRNA signature
CRC	Colorectal cancer
IAMIPS	immune-associated miRNA prognostic signature
PTM	peritoneal metastasis
LM	Leptomeningeal metastasis
BC	breast cancer
TCGA	The Cancer Genome Atlas
GEO	Gene Expression Omnibus
PCa	Prostatic cancer
BPH	benign prostatic hyperplasia
PSA	prostate specific antigen
ADT	androgen-deprivation therapy
CRPC	castration-resistant prostate cancer
BF	biochemical disorder
CF	clinical failure
PFS	progression-free survival
RFS	relapse free survival
BRC	biochemical relapse
RP	radical prostatectomy
SERS-LFA	surface-enhanced Raman scattering-lateral flow assay
TEP	tumor-educated platelet
SEVs	small extracellular vesicles
EV	extracellular vesicles
EBC	exhaled breath condensate
ACP	acid phosphatase
ER	estrogen receptor
PR	progesterone receptor
DRE	digital rectal examination
FOBT	fecal occult blood test
